# A Clinical Contribution

**Published:** 1884-06

**Authors:** Mahlon Preston

**Affiliations:** Norristown, Pa.


### A CLINICAL CONTRIBUTION.

Mahlon Preston, M. D., Norristown, Pa.

I was recently placed under the necessity of selecting a remedy for a case belonging to a class which I had seen prove fatal in a number of instances, and for which the best proven of our drugs had failed to give satisfactory results.

The symptoms presented were as follows, and the combination was one which I have learned to dread.

Extremely dry mouth, with thirst which gave but partial and momentary relief; thick, dry coating on the tongue, yellowish and dark, forming a crust which rendered it almost immovable.

Dryness of the pharynx extreme and painful, with soreness as if it would crack, impeding and finally obstructing deglutition.

Dry scaly crusts covered the roof of the mouth, which water scarcely served to dissolve or soften.

Mucus collection in the pharynx, very difficult to detach or wash away by gargling; it produced a horrid taste which disgusted the patient, particularly when he attempted to take food. The mucus was slimy, glutinous, and frothy.

Patient imagines himself able to take some food, a sort of hungry feeling and a conception that the morsel would be agreeable, but when it reaches the pharynx it is immediately expelled from a horrid nausea arising from the lodgment of mucus there.

Constant necessity to hawk and spit, to clear out the pharynx; expectoration of considerable quantities of the glutinous, frothy mucus causing gagging; taste bitterish and putrid.

Indescribable feeling of distress in the epigastric region, including space over transverse colon; this place seems sunken in; great weakness; it was referred principally to this locality, with respiration audible, slow, and grunting.

Pinched countenance; feeble, rapid pulse; cold extremities, bloodless fingers.

Urine moderate in quantity, high colored; passed freely but rarely; loaded with red sand. Stool rare and clay-colored.

These symptoms presented in a tall, robustly developed gentleman of about seventy, and had deprived him of strength and flesh, in three or four weeks' time, till he was but a shadow of his usual self at the time I was invited to see the case; had been during this time under allopathic treatment. Ars., Puls., Phos., Lycop., Sulph., were resorted to in about the order named but failed to produce any beneficial result.

A careful review of the pathogenesis of Myrica cirif. in *Allen* led me to believe there might be a hope, and it was given in water, a dose every four hours. After the fourth dose a remarkable change came over the existing condition, mucus began to be more easily detached from the pharynx, and it soon ceased to form, nausea disappeared, and appetite came; urine became light-colored and considerably profuse without any deposit.

In a week's time all serious phases had vanished and the patient began to desire to resume his usual habits and to venture out.

Detained by the inclement weather, he was, however, able to enjoy a ride in ten days and to eat like a plowman.

My speculations have not led me to any satisfactory diagnosis of this case, but I have seen within two years two persons die in precisely the same circumstances where the urine finally became excessively loaded with albumen, speedily followed by general paralysis, and I am unable to withold the conviction that Myrica cirif., if resorted to in time, might have saved them; at all events, I shall not soon forget the efficient service it has rendered me, nor hesitate to depend on it in the future for like symptoms.

